# Multicenter cross-sectional study on sexual violence among university students: victims, perpetrators, and alcohol use

**DOI:** 10.3389/fpubh.2026.1647953

**Published:** 2026-02-26

**Authors:** Alba Berenguer-Simon, David Ballester-Ferrando, Zaira Reyes-Amargant, Paola Galbany-Estragués, Elena Maestre-González, Eva María Garrido-Aguilar, Maria Dolors Burjalés-Marti, Rebeca Gomez-Ibañez, Eva Serrat-Graboleda, Dolors Rodríguez-Martín, Concepció Fuentes-Pumarola

**Affiliations:** 1 Health, Gender and Aging Research Group, Department of Nursing, University of Girona, Girona, Spain; 2 Health Assistance Institute, Girona, Spain; 3 Catalan Health Institute, Girona, Spain; 4 Department of Fundamental and Clinical Nursing, Faculty of Nursing, University of Barcelona, Barcelona, Spain; 5 AFIN Research Group and Outreach Centre, Autonomous University of Barcelona, Barcelona, Spain; 6 GENI Research Group in Gender, Identity and Diversity, University of Barcelona, Barcelona, Spain; 7 Department of Public Health, Mental Health and Maternal and Child Health Nursing, Faculty of Nursing, University of Barcelona, Barcelona, Spain; 8 Department of Nursing, Advanced Nursing Research Group, Rovira I Virgili University, Tarragona, Spain; 9 Nursing Department, Faculty of Medicine, University Autonomous Barcelona, Barcelona, Spain

**Keywords:** alcohol drinking in college, leisure, peer influence, sexual violence, students, universities

## Abstract

**Objective:**

This study aims to estimate the prevalence of sexual violence among university students (identification of sexual violence behaviors, self-recognition as victims and perpetrators) and its relationship with alcohol consumption and other associated factors.

**Methods:**

Descriptive cross-sectional multicenter study of students from six public universities in Catalonia, north-eastern Spain. Sexual violence has been categorized into three levels of severity, ranging from least to most severe.

**Results:**

2,803 students participated (72.5% women). Regarding sexual violence victimization, 79% of respondents had experienced level 1, 59.8% level 2, and 7.5% level 3. Concerning perpetration, 15.6% had perpetrated level 1, 2.5% level 2 and 0.3% level 3. Sexual violence identification was high, with females identifying more situations than males (*p* < 0.001). 39.6% of students had felt pressured to drink alcohol and 65% had experienced alcohol-related blackouts in the previous year. Men consumed more alcohol than women (*p* < 0.001). Sexual violence perpetration was associated with males, students in 4th to 6th grade, pressure to consume alcohol, alcohol-related blackouts and alcohol consumption. Sexual violence victimization was associated with females, pressure to consume alcohol, alcohol-related blackouts and alcohol consumption.

**Conclusion:**

This study identifies a high prevalence of sexual violence among university students, consistent with international findings and signaling a concerning trend. The results also show a strong link between sexual violence and alcohol consumption in university leisure contexts. Existing evidence indicates that alcohol facilitates rather than causes sexual violence, creating circumstances in which sexual victimization becomes more likely. The study highlights the need for prevention strategies and educational programs addressing sexual violence and reducing its associated risk factors among university students.

## 1 Introduction

Sexual Violence (SV) is defined by the World Health Organization (WHO) as *“any sexual act, attempt to obtain a sexual act, or other act directed against a person’s sexuality using coercion, by any person regardless of their relationship to the victim, in any setting. It includes rape, defined as the physically forced or otherwise coerced penetration of the vulva or anus with a penis, other body part or object”* (1). SV is widespread in university environments and its prevalence among university students is a matter of considerable concern (2). Research indicates that SV in this context, which predominantly affects women, manifests in various forms. However, few studies make distinctions between different levels of severity. Moreover, reported prevalence rates vary according to the country or region in which the studies are conducted. A meta-analysis (3) examining studies from 21 countries reports a prevalence of sexual assault of 17.5% for women, 7.8% for men and 18.1% for transgender and gender-diverse individuals. In the United Kingdom, the prevalence of sexual harassment is reported to be 52.7%, with 20.5% of respondents experiencing at least one act of attempted or forced sexual touching or rape (4). In the European context, a study conducted in three universities (two in the Netherlands and one in Belgium) identified a prevalence of 56% for sexual assaults, with 65% of women reporting having experienced sexual assault. The study also indicated that the incidents reported were mostly unwanted sexual contact (touching) perpetrated by unknown men aged between 18 and 35 years (5). In the United States of America (USA), 17% of female university students report having experienced at least one instance of unwanted sexualized touching since enrolling, while 4.2% of men students reported similar experiences. Approximately 9% of female students have reported experiencing penetrative sexual assault during their time at university (6). A study (7) conducted in Spain found that 20.8% of female students experienced intimate partner SV. Specifically, 5% felt coerced into sex “just to avoid giving explanations,” 11.6% endured unwanted and unpleasant touching, 9.9% were pressured to perform specific sexual acts and 4.6% were forced to undress against their will.

Despite the high prevalence of sexual violence (SV) in university settings, a study conducted in Spain highlighted the difficulties students face in recognizing situations of SV (8). This factor, combined with the leisure contexts in which many of these incidents occur (9) and the role of alcohol consumption is frequently implicated in SV incidents (10). In the United States, 49.6% of young adults reported alcohol use in the past month (11). Alcohol consumption is a key predictive factor for SV perpetration, with perpetrators often reporting higher frequencies and quantities of alcohol use than non-perpetrators (12). Approximately half of SV incidents among university students occur when the perpetrator, the victim, or both have consumed alcohol (13).

In this context, alcohol-related blackouts (ARBs), defined as “gaps in a person’s memory for events that occurred while they were intoxicated,” play an important role. More than half of young adults and university students who drink report on experiencing ARBs (14). ARBs are believed to be involved in many SV incidents, particularly those in which either victims or perpetrators have consumed large amounts of alcohol (15).

In the context of sexual violence in nightlife, peer pressure also contributes significantly to alcohol consumption among university students, particularly men, and often leads to substantial increases in drinking behavior (16).

In light of this situation, this study aimed to estimate the prevalence of sexual violence among university students, focusing on the identification of sexual violence behavior’s and self-recognition as victims and perpetrators, and to analyze the relationship between sexual violence and alcohol use, as well as other associated factors.

## 2 Method

### 2.1 Study design and setting

This cross-sectional, descriptive, non-randomized study utilized a self-administered questionnaire and was carried out between February 2018 and June 2020.

The study population consisted of 105,257 undergraduate students from six public universities in Catalonia (north-eastern Spain): Autonomous University of Barcelona, University of Barcelona, University of Girona, Rovira and Virgili University in Tarragona, University of Lleida and University of Vic.

### 2.2 Sample

Based on a 20% prevalence rate for sexual abuse and assault reported in previous studies in Spain (17–19), the sample size was calculated with a 95% confidence level, a 5% precision and a design effect of 1, yielding an estimated sample size of 1,455. The inclusion criteria required participants to be present in the classroom on the day of data collection and to complete the questionnaire. The questionnaire was distributed to 3,222 students, with 2,803 ultimately participating in the study (87%). Of these, 52.9% (*n* = 1,484) were from the Autonomous University of Barcelona, 19.3% (*n* = 540) from the University of Barcelona, 10.3% (*n* = 288) from Rovira and Virgili University, 8.6% (*n* = 242) from the University of Girona, 5.7% (*n* = 169) from the University of Vic, and 3.2% (*n* = 160) from the University of Lleida.

Table 1 outlines the sociodemographic characteristics of the participants. Among the participants, 72.5% were women, with a mean age of 20.50 years (SD: 1.77).

**Table 1:** Sociodemographic data (*N* = 2,803).

Age, mean (SD)	20.50 (1.77)
Sex, *n* (%)	
Female	2,031 (72.5)
Male	772 (27.5)
Study Area, *n* (%)	
Arts and humanities	357 (12.7)
Social and legal sciences	921 (32.9)
Sciences	488 (17.4)
Health sciences	883 (31.5)
Engineering and architecture	154 (5.5)
Academic year, *n* (%)	
1st	770 (27,5)
2nd	695 (24.8)
3rd	625 (22.3)
4th	532 (19)
5th	136 (4.9)
6th	45 (1.6)

### 2.3 Instrument

The survey comprised an *ad hoc* questionnaire divided into two sections. The first section gathered (a) demographic data, including age, sex (with respondents self-identifying as male or female) and academic year (ranging from first to sixth year), and (b) data on alcohol consumption (measured in Spanish Standard Drinks ─SSD─, where 1 SSD corresponds to 10 grams of alcohol), alcohol-related blackouts in the previous year and pressure to consume alcohol. The second section assessed SV behavior’s as follows: (1) “To make obscene gestures or looks”; (2) “To insinuate oneself and make the other person uncomfortable”; (3) “To make unwanted touches”; (4) “To engage in intimidating conversations for the other person”; (5) “To engage in intimidating cornering”; (6) “To pressure someone to perform sexual acts that were not consummated”; (7) “To pressure someone to perform unwanted sexual acts that were consummated”; (8) “To force someone to perform unwanted sexual acts that were consummated.”

Regarding these behaviors’, students were asked to identify those they considered to constitute SV by responding to the prompt: “Indicate which behavior’s you consider to be SV.” They were also questioned about their personal experiences, both as victims (“While partying, have you experienced any of these situations?”) and as perpetrators (“While partying, have you caused any of these situations?”).

These SV behaviors’ were categorized into three levels of severity. Level 1 included to low-intrusion behavior’s involving non-physical sexual harassment as: “To make obscene gestures or looks” and “To insinuate oneself and make the other person uncomfortable.” Level 2 encompassed situations where pressure or coercion is exerted, but the victim is still able to avoid the unwanted sexual act as: “To make unwanted touches”; “To engage in intimidating conversations for the other person”; “To engage in intimidating cornering” and “To pressure someone to perform sexual acts that were not consummated.” Level 3 comprised situations in which the coercion results in the actual execution of unwanted sexual acts, implying a higher degree of physical violation and invasion of personal integrity as: “To pressure someone to perform unwanted sexual acts that were consummated,” refers to situations in which the victim suffers psychological, emotional, or social coercion (manipulation, threats of non-physical consequences, persistent pressure); and “To force someone to perform unwanted sexual acts that were consummated” refers to situations where the perpetrator uses physical force, physical restraint, or threats of immediate physical harm to overcome the victim’s resistance.

### 2.4 Data collection

University faculties collaborated with researchers to organize the administration of the questionnaire in classrooms. Researchers briefed students on the study’s objectives, ensured anonymity, and obtained informed consent. The questionnaire was provided in electronic format via QR code and in paper form to safeguard confidentiality. Completion time was estimated at approximately 10 min. The study was approved by the University Bioethics Committee and conducted in accordance with Good Clinical Practice, the Code of Good Research Practice, and the Declaration of Helsinki. Permissions were obtained from faculty deans. All participants provided informed consent, and data protection requirements under Law 3/2018 were met. Anonymity and confidentiality were ensured throughout the process.

### 2.5 Data analysis

A univariate descriptive analysis was conducted for all variables. Continuous variables were described using mean, standard deviation (SD), median, and interquartile range (IQR), whereas categorical variables were expressed as percentages. Bivariate analysis was performed used Pearson’s chi-square test or Fisher’s exact test, and logistic regression was applied to variables that were statistically significant. Results were deemed statistically significant at *p* < 0.05 with a 95% confidence interval (CI). All analyses were carried out using SPSS for Windows, version 27 (SPSS Inc., Chicago, IL, USA).

### 2.6 Rigor

This study was conducted following the STROBE checklist for study design, data collection, analysis, and publication. A pilot interview was conducted beforehand to ensure the proper functioning of the interview process.

## 3 Results

**Figure 1:**
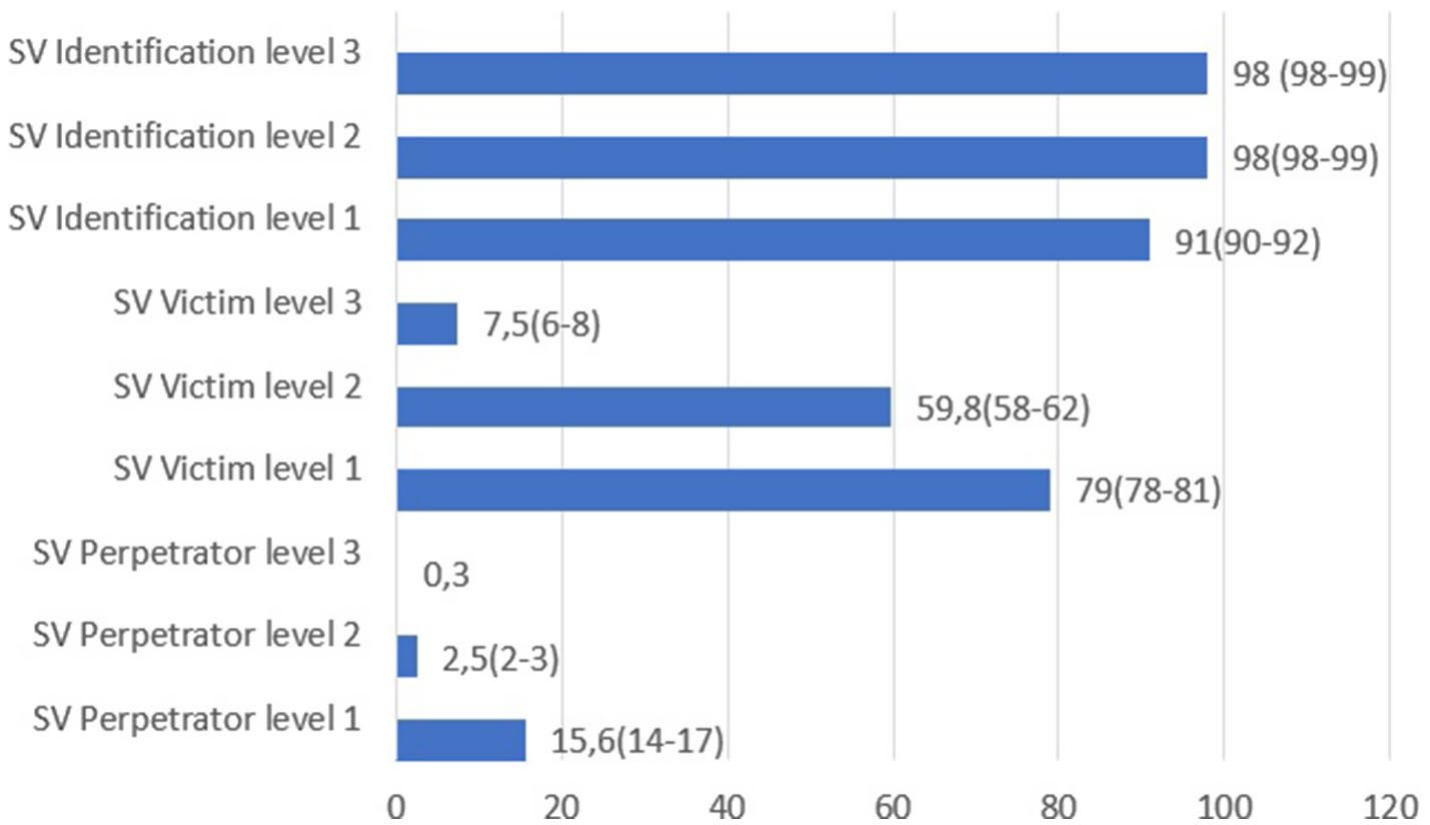
Prevalence of SV identification, victims, and perpetrators. Prevalence estimates (95% confidence interval) (*N* = 2,803).

Figure 1 illustrates the prevalence of SV and the 95% confidence interval. A total of 79% of participants (*n* = 2,215) identified themselves as victims of Level 1 SV (low-intrusion behaviors’ involving non-physical sexual harassment), including 91.1% of women (*n* = 1,851) and 47.2% of men (*n* = 364). Among women, 71.8% (*n* = 1,458) reported experiencing Level 2 SV (situations where pressure or coercion is exerted but the victim is still able to avoid the unwanted sexual act), and 9.3% (*n* = 189) reported Level 3 SV (situations in which coercion results in the actual execution of unwanted sexual acts, representing a higher degree of physical violation and invasion of personal integrity).

In addition, 28.1% of men (*n* = 217) and 10.9% of women (*n* = 221) identified themselves as perpetrators of Level 1 SV. Although self-reported rates of involvement in SV are high across levels, women appear to recognize and identify SV more readily than men (Table 2).

**Table 2:** Prevalence and relationship of sexual violence by sex.

Variable	Sex *n* (%)		*p* value^*^
	Male	Female	
SV identification level 1	667 (86.4)	1,877 (92.4)	<0.001
SV identification level 2	743 (96.2)	2,010 (99)	<0.001
SV identification level 3	747 (96.8)	2,013 (99.1)	<0.001
SV perpetrator level 1	217 (28.1)	221 (10.9)	<0.001
SV perpetrator level 2	44 (5.7)	26 (1.3)	<0.001
SV perpetrator level 3	3 (0.4)	5 (0.2)	0.814
SV victim level 1	364 (47.2)	1,851 (91.1)	<0.001
SV victim level 2	219 (28.4)	1,458 (71.8)	<0.001
SV victim level 3	20 (2.6)	189 (9.3)	<0.001

*Chi square.

The relationship between SV, study area and academic year is detailed in Table 3. Notably, students in the 4th to 6th academic year identify more situations of SV and are more likely to recognize themselves as both victims and perpetrators compared to students in earlier academic years (*p* < 0.005).

**Table 3:** Relationship of sexual violence with study area and academic year.

Variable	Study area						Academic year			
	Arts and Humanities *n* (%)	Social and legal sciences *n* (%)	Sciences *n* (%)	Health sciences *n* (%)	Engineering and architecture *n* (%)	*p*-value	1st to 3rd *n* (%)	4rd to 6th *n* (%)	*p*-value	Cramer’s V
SV identification level 1	335 (93.8)	819 (88.9)	437 (89.5)	814 (92.2)	139 (90.3)	0.029	1,880 (90.0)	664 (93.1)	0.011	0.048
SV identification level 2	352 (98.6)	901 (97.8)	479 (98.2)	871 (98.6)	150 (97.4)	0.632	2,048 (98.0)	705 (98.9)	0.122	0.029
SV identification level 3	351 (98.3)	904 (98.2)	481 (98.6)	874 (99)	150 (97.4)	0.501	2,054 (98.3)	706 (99.0)	0.165	0.026
SV perpetrator level 1	54 (15.1)	150 (16.3)	69 (14.1)	137 (15.5)	28 (18.2)	0.743	286 (13.7)	152 (21.3)	<0.001	0.092
SV perpetrator level 2	9 (2.5)	26 (2.8)	14 (2.9)	18 (2)	3 (1.9)	0.796	42 (2.0)	28 (3.9)	0.005	0.054
SV perpetrator level 3	1 (0.3)	4 (0.4)	1 (0.2)	2 (0.2)	0 (0)	0.784	5 (0.2)	3 (0.4)	0.427*	0.015
SV victim level 1	286 (80.1)	745 (80.9)	375 (76.8)	691 (78.3)	118 (76.6)	0.350	1,624 (77.7)	591 (82.9)	0.003	0.055
SV victim level 2	230 (64.4)	540 (58.6)	296 (60.7)	522 (59,1)	89 (57.8)	0.370	1,216 (58.2)	461 (64.7)	0.002	0.058
SV victim level 3	25 (7)	80 (8.7)	34 (7)	62 (7)	8 (5.2)	0.451	147 (7.0)	62 (8.7)	0.145	0.028

*Fisher.

Alcohol consumption, measured in Spanish Standard Drinks (SSD), where 1 SSD equals 10 grams of alcohol, was notably high, with a mean of 18.84 (SD: 14.42), a median of 14.50, and an Interquartile Range (IQR) of 16.50. Furthermore, 39.6% of the students reported feeling pressured to drink alcohol, and 65% reported experiencing ARBs in the past year. Male students consumed more alcohol than female (*p* < 0.001), while students of Social and Legal Sciences reported the highest levels of alcohol consumption. No statistically significant differences were identified between alcohol consumption and academic year (Supplementary Material S1).

The relationship of SV with alcohol consumption variables is presented in Table 4. Participants who self-identified as either perpetrators or victims of SV, irrespective of severity, reported experiencing higher pressure to consume alcohol (*p* < 0.05) and a greater frequency of ARBs (*p* < 0.05). Furthermore, higher levels of alcohol consumption were linked to self-identification as a victim (levels 2 and 3) or as a perpetrator (levels 1 and 2).

**Table 4:** Relationship of sexual violence with sociodemographic and alcohol consumption variables.

Variable	Pressure to consume alcohol *n* (%)		*p* value*	Alcohol-related blackouts *n* (%)		*p* value*	Spanish standard drink median (SD)		*p* value**
	Yes	No		Yes	No				
SV perpetrator level 1	240 (21.6)	197 (11.7)	<0.001	347 (19.1)	88 (9)	<0.001	Yes	18.00 (19.00)	<0.001
							No	13.50 (13.00)	
SV perpetrator level 2	42 (3.8)	28 (1.7)	<0.001	59 (3.2)	11 (1.1)	<0.001	Yes	17.50 (13.50)	0.003
							No	14.00 (16.50)	
SV perpetrator level 3	6 (0.5)	2 (0.1)	0.042	8 (0.4)	0 (0)	0.009	Yes	18.00 (43.00)	0.192
							No	14.50 (16.50)	
SV victim level 1	929 (83.7)	1,284 (76)	<0.001	1,518 (83.6)	693 (70.8)	<0.001	Yes	14.50 (16.00)	0.095
							No	13.59 (17.50)	
SV victim level 2	736 (66.3)	940 (55.6)	<0.001	1,234 (68)	441 (45)	<0.001	Yes	15.00 (16.50)	<0.001
							No	13.50 (15.50)	
SV victim level 3	133 (12)	76 (4.5)	<0.001	179 (9.9)	30 (3.1)	<0.001	Yes	17.50 (18.00)	0.002
							No	14.00 (16.00)	

*Chi square. **U Mann–Whitney.

Table 5 presents the regression analysis of self-recognition as victims of SV and self-recognition as perpetrators by sex, age, academic year, SSD, pressure to consume alcohol, and ARBs. The results of the logistic regression analysis indicate that the self-recognition as perpetrators is linked to males (levels 1 and 2), students in their 4th to 6th academic year (levels 1 and 2), pressure to consume alcohol (levels 1 and 2), ARBs (levels 1 and 2), and alcohol consumption (level 1). Conversely, self-recognition as victims is associated with being female, pressure to consume alcohol, ARBs and alcohol consumption, across all levels of severity.

**Table 5:** Regression analysis of SV victim, perpetrator: sex, age, academic year, pressures to consume alcohol, alcohol-related blackouts and Spanish Standard drink.

Variable		SV perpetrator level 1		SV perpetrator level 2		SV perpetrator level 3	
		OR	95% CI	OR	95% CI	OR	95% CI
Sex	Female	Reference					
	Male	3.07*	2.41–3.90	4.28*	2.56–7.17	1.03	0.19–5.39
Age		1.09*	1.00–1.18	0.94	0.78–1.13	0.92	0.52–1.63
A. year	1st to 3rd	Reference					
	4rd to 6th	1.42*	1.04–1.93	2.39*	1.22–4.68	2.28	0.31–16.38
Pressure	No	Reference					
	Yes	2.07*	1.63–2.63	2.01*	1.20–3.36	3.76	0.72–19.56
ARBs	No	Reference					
	Yes	1.84*	1.37–2.46	3.01*	1.40–6.45	54,26 × 10^5^	0.00
SSD		1.01*	1.01–1.02	1.00	0.99–1.02		

Variable		SV victim level 1		SV victim level 2		SV victim level 3	
		OR	95% CI	OR	95% CI	OR	95% CI
Sex	Male	Reference					
	Female	15.66*	12.11–20.24	8.29*	6.66–10.33	4.83*	2.80–8.31
Age		1.07*	0.99–1.17	1.02	0.95–1.09		
A. Year	1st to 3rd	Reference					
	4rd to 6th	0.90	0.63–1.28	1.01	0.76–1.32	9.30	0.60–1.42
Pressure	No	Reference					
	Yes	2.02*	1.55–2.64	1.89*	1.54–2.32	3.24*	2.35–4.46
ARBs	No	Reference					
	Yes	2.00*	1.53–2.60	2.19*	1.77–2.70	2.76*	1.77–4.32
SSD		1.00*	1.00–1.01	1.01*	1.00–1.02	1.01*	1.00–1.02

*p < 0.05. ARBs, Alcohol-related blackouts; SSD, Spanish standard drink = 10 grams of alcohol.

## 4 Discussion

The objectives of this study were to assess the prevalence of SV among university students, including identifying SV behaviors’ and self-recognition as victims and perpetrators, and to explore the relationship between SV and alcohol consumption, pressure to consume alcohol and ARBs.

In relation to SV identification, although nearly all students (98%) identified levels 1 (low-intrusion behaviors’ involving non-physical sexual harassment) and 2 (situations where pressure or coercion is exerted, but the victim is still able to avoid the unwanted sexual act) as SV, the identification of level 1 was slightly lower (91%). It is well-established that behavior’s involving greater violence are more likely to be identified as SV (8, 20). Moreover, the difficulty in identifying SV is particularly pronounced among men (8). This difficulty among young Spanish men may be attributed to persistent myths about sexual aggression, which are more prevalent among men than women. Such myths trivialize the SV experienced by women; for example, the belief that “when a man urges his female partner to have sex, it cannot be called rape” (21). Despite this trivialization, women experience significant negative consequences, such as feelings of disrespect, disgust, anger, shame or rape, particularly in cases of violence involving physical contact (levels 2 and 3) (22).

In terms of self-recognition as SV victims, our findings are consistent with a study conducted among Norwegian university students (23), which demonstrated that less severe forms of SV have a higher prevalence of victimization compared to more severe forms. Regarding the prevalence of self-recognition as level-2 SV victims (59.8%), similar results have been found in a European study (56%) (5). Additionally, women reported experiencing SV more frequently than men.

While only a small number of participants in our study admitted to perpetration, previous research involving university students has reported even lower prevalence rates. Notably, a higher proportion of perpetrators were identified as male (24).

In relation to alcohol consumption participants in our study reported consuming an average of 18.84 Spanish Standard Drinks (1 SSD = 10 g of alcohol) when they go out partying, equivalent to approximately nine mixed drinks. In recent years, binge drinking, defined as consuming 5 or more drinks within a two-hour period for men and 4 or more drinks for women, has become a prevalent drinking pattern among young people and adolescents (25). Our study did not specify a time frame; nevertheless, the levels of alcohol consumption observed were remarkably high. According to the WHO (1), Europe has the highest prevalence of alcohol consumption globally. The findings align with previous research indicating that men tend to consume more alcohol than women (26). Moreover, it is well-documented that young people often engage in heavy drinking during leisure time, as it is perceived to facilitate interpersonal relationships (27).

Regarding pressure to consume alcohol and ARBs, approximately 40% of the students in our sample reported experiencing pressure to drink. Previous research on university students has documented lower prevalence rates (9, 28). Consistent with similar findings among Spanish nursing students (9), 65% of the students in our sample reported experiencing ARBs within the past year. In contrast, studies conducted with American university students have reported lower prevalence rates, around 40% (26).

Numerous studies have emphasized the link between alcohol consumption, leisure and SV (29–31). Our research found that students identified as either victims or perpetrators of SV reported greater pressure to consume alcohol, experienced more ARBs, and displayed higher levels of alcohol consumption. It is widely acknowledged that social relationships are closely tied to alcohol consumption, with university groups often using alcohol to foster social bonds and enhance group cohesion. Challenging this cultural norm requires confronting deep-rooted traditions and significant peer pressure (32). Several studies have further associated excessive alcohol consumption, five or more drinks in a single day, with the perpetration of SV among young men (33, 34), with alcohol being perceived by perpetrators as a positive facilitator in sexual encounters due to its disinhibitory effects and its role in facilitating interpersonal relations (35).

This study identified a relationship between being female, experiencing pressure to consume alcohol, encountering ARBs, engaging in high levels of alcohol consumption, and being a victim of SV across all levels of severity. Other studies support these findings, demonstrating that women in leisure settings and alcohol consumption suffer from level 1 and 2 SV (verbal and physical harassment, cornering and groping) (35).

## 5 Strengths and limitations

A notable strength of this study lies in its multi centre design and high participation rate, which enable more representative data collection and enhance external validity.

Furthermore, the study’s emphasis on identifying sexual violence behavior’s and situations, as well as self-recognition as sexual violence perpetrators, rather than solely focusing on victimization, is particularly commendable.

However, the study has several limitations. These include the inability to verify the accuracy of survey responses, the inherent restriction of establishing definitive cause-and-effect relationship due to the cross-sectional study design, and the reliance on a convenience sample, which constrains the generalizability of the findings to the wider student population.

Additionally, the study did not consider the gender identity and sexual orientation of participants.

It is also important to highlight the lack of consensus regarding the classification of sexual violence, which complicates comparisons across studies.

## 6 Conclusion

This study reveals a high prevalence of sexual violence among university students, consistent with international research and reflecting a persistent and concerning trend. The findings also show a clear association between sexual violence, alcohol consumption, and peer pressure within university leisure settings. However, existing evidence indicates that alcohol does not directly cause sexual violence but rather acts as a facilitator for individuals who already hold harmful attitudes and beliefs, thereby increasing the likelihood of situations in which sexual victimization may occur.

These findings highlight the pressing need for targeted prevention strategies aimed at addressing key risk factors. Moreover, the study emphasizes the importance of educational programs in raising awareness, promoting respect and accountability, and equipping students with the necessary skills to navigate social and leisure environments safely. Such initiatives are essential for reducing instances of sexual violence and fostering a safer, more inclusive university community.

## Data Availability

The raw data supporting the conclusions of this article will be made available by the authors, without undue reservation.
