# Supplementary material for: Multicenter cross-sectional study on sexual violence among university students: victims, perpetrators, and alcohol use
Source: Front Public Health. 2026 Feb 26;14:1647953. doi: 10.3389/fpubh.2026.1647953 (PMC12979539; doi:10.3389/fpubh.2026.1647953)
Supplement: Supplementary file 1 [file Table_1.docx]

Supplementary Material

**Supplementary material 1**

*Alcohol consumption by sex, study area and academic year*

|  | | **Spanish Standard Drink**  Median (IQR) | **P value** |
| --- | --- | --- | --- |
| **Sex** | Male | 16.00 (18.60) | **<.001*** |
|  | Female | 14.00 (15.50) |  |
| **Study Area** | Arts and Humanities | 15.00 (16.5) | **<.001**** |
|  | Social and Legal Sciences | 16.00 (17.50) |  |
|  | Sciences | 14.00 (18.30) |  |
|  | Health Sciences | 13.00 (14.10) |  |
|  | Engineering and architecture | 13.00 (17.05) |  |
| **Academic Year** | 1st to 3rd | 14.50 (17.50) | .424* |
|  | 4rd to 6th | 14.50 (13.50) |  |

Spanish Standard Drink (SSD): 1 SSD= 10 grams of alcohol

* U Mann-Whitney

** Kruskal-Wallis test, significant differences are only observed between Health Sciences and Social and Legal Sciences
